# Adapting a Dutch Web-Based Intervention to Support Family Caregivers of People With Dementia in the UK Context: Accelerated Experience-Based Co-Design

**DOI:** 10.2196/52389

**Published:** 2024-05-22

**Authors:** Fiona Scheibl, Lizzy Boots, Ruth Eley, Christopher Fox, Fergus Gracey, Karen Harrison Dening, Jan Oyebode, Bridget Penhale, Fiona Poland, Gemma Ridel, Juniper West, Jane L Cross

**Affiliations:** 1 Institute of Applied Health Research College of Medical and Dental Sciences Birmingham United Kingdom; 2 Department of Psychiatry and Neuropsychology Alzheimer Center Limburg, Mental Health and Neuroscience Research Institute Maastricht University Medical Centre+ Maastricht Netherlands; 3 Together Everyday in Dementia Liverpool United Kingdom; 4 College House University of Exeter Devon United Kingdom; 5 Faculty of Medicine and Health Sciences University of East Anglia Norwich United Kingdom; 6 Dementia UK London United Kingdom; 7 Centre for Applied Dementia Studies Faculty of Health Studies University of Bradford Bradford United Kingdom; 8 Research and Development Norfolk and Suffolk NHS Foundation Trust Norwich United Kingdom

**Keywords:** adaptation, caregivers, dementia, intervention, web-based resources, United Kingdom, co-design, web-based intervention, support, carer, caregiver, family carer, community-based, services, dementia, web-based support, staff, self-help, web-based

## Abstract

**Background:**

Around 700,000 family caregivers provide unpaid care for 900,000 people living with dementia in the United Kingdom. Few family caregivers receive support for their own psychological needs and funding for community respite services has declined. These trends are seen across Europe as demographic and budgetary pressures have intensified due to public spending cuts arising from the 2008 financial crisis and the COVID-19 pandemic. The World Health Organization has prioritized the need to expand the provision of support for caregivers and families of people with dementia by 2025. Web-based interventions have the potential for development as they require modest investment and can be accessed by family caregivers at home. Further cost benefits can be realized by adapting existing interventions with demonstrated effectiveness for new contexts. This paper reports initial findings from the CareCoach study, which is adapting Partner in Balance (PiB), a web-based coaching intervention developed in the Netherlands, for family caregivers in the United Kingdom.

**Objective:**

This study aims to work with unpaid family caregivers and staff in adapting the Dutch web-based support tool PiB to improve its acceptability and usability for use in the United Kingdom.

**Methods:**

Accelerated Experience-Based Co-Design (AEBCD) was used with caregivers, staff, and core stakeholders. Interviews, workshops, and stakeholder consultations were conducted. Data were analyzed iteratively. Recommendations for the redesign of PiB for use across the United Kingdom were adjudicated by the study Adaptation Working Party.

**Results:**

Sixteen caregivers and 17 staff took part in interviews. Thirteen caregivers and 17 staff took part in workshops. Most (n=26) participants were White, female, and retired. All except 4 caregivers (2 male and 2 female) found the PiB’s offer of web-based self-help learning acceptable. Caregivers identified complexity and lack of inclusivity in some wording and video resources as problematic. The staff took a stronger perspective on the lack of inclusivity in PiB video resources. Staff and caregivers coproduced new inclusive wording and recommended creating new videos to adapt PiB for the UK context.

**Conclusions:**

AEBCD methods facilitated the engagement of caregivers and staff and advanced the adaptation of the PiB complex intervention. An important addition to the AEBCD method in this process was the work of an Adaptation Working Party, which adjudicated and agreed to new wording where this could not be established in consultation with caregivers and staff.

**Trial Registration:**

ISRCTN Registry ISRCTN12540555; https://doi.org/10.1186/ISRCTN12540555

## Introduction

### Background

Around 700,000 family caregivers support 900,000 people living with dementia [1]. Most unpaid caregivers are female [2] providing care as spouses or partners or adult daughters, although increasing numbers of sons, siblings, grandchildren, and friends care for an older person living with dementia. Males older than 66 years provide the most hours of unpaid care (50+) a week while females in the age group of 31-45 years provide the highest levels of intense unpaid care reflecting the wider social trend for more people of working age to combine paid work with caring which can lead to increased risk of poor health and burnout [2]. Throughout the discussion presented in this paper, we use the terms “family caregiver,” “caregiver,” or “unpaid caregiver” as equivalent terms.

The unpaid assistance that family caregivers provide is estimated to save the UK government £13.9 billion (1 GB £=US $1.266502) each year adding vital support to the formal care system [3] and current UK health and social care policy is premised on its continued provision [4]. National Institute for Health and Care Excellence (NG97) guidance recommends expanding support for caregivers in the United Kingdom through the provision of psycho-education, dementia education, and advice on physical and mental health to help build individual personal strategies to cope with the behavioral and communication challenges of the caring role [5].

A majority (64%) of unpaid caregivers report lower levels of social contact than they would like, placing strain on family relationships [6,7]. More than a quarter report having poor mental health [8] while 75% of those in paid work report high levels of worry about juggling care and work [9]. Caregivers in minority ethnic groups face additional challenges associated with cultural and community norms, language, literacy, and stigma which increase isolation and depression [6].

Few family caregivers receive support for their own psychological needs [9] due in part to the lack of available skilled therapists [10]. Publicly funded community-based services (such as daycare centers) have declined by 30% since 2005 despite rapid growth in the number of people with dementia [11] placing an additional burden on caregivers. These trends are seen across Europe as demographic and budgetary pressures have intensified due to public spending cuts arising from the impacts of the 2008 financial crisis [11] and the COVID-19 pandemic.

### Potential of Web-Based Interventions to Offer Cost-Effective Support for Family Caregivers

Web-based interventions require modest investment, can be accessed at a time chosen by the participant [12], may appeal to caregivers who prefer to access services at home, and avoid costs associated with traveling [6]. They can be tailored to deliver a mix of self-support skills and education, are cost-effective, and have scalability [12]. While robust evidence on the effectiveness of web-based interventions for family caregivers of people with dementia is limited [13,14] web-based self-administered support tools can reinforce the positive aspects of caring and improve resilience and overall well-being [6,15] and caregivers value the easy access [16]. The proportion of people with caring responsibilities accessing the internet in the United Kingdom has grown in recent years [17]; however, inequities in access due to poor technological skills and access to internet resources persist and may compromise inclusivity [6,18].

### Key Issues in the Cultural Adaptation of Interventions

Ensuring a good fit between the cultural identity of the target community and the content of web-based interventions is important to their effectiveness [19,20]. Adapted interventions that prioritize cultural congruence have shown better outcomes [21,22]. While some commonalities exist in dementia care and caregivers’ needs across the boundaries of culture, adaptation to specific contexts requires adjustments to language, communication, and wider norms and practices [23,24]. Familiar, accessible language is identified as particularly important for engagement with web-based interventions [25,26].

### Adapting Partner in Balance for the UK Context

This paper reports research undertaken as part of the CareCoach Program (Program Grant for Applied Research [NIHR201076]) [27], which used Experience-Based Co-Design methods to work toward the creation of a web-based coached intervention for caregivers of people with dementia, referred to throughout the paper as “CareCoach,” based on Partner in Balance (PiB) [28] developed in the Netherlands.

PiB is a web-based self-support tool for caregivers of people with a recent diagnosis of mild to moderate dementia. PiB was selected for adaptation in the CareCoach program because it reported significant positive effects on caregiver self-efficacy, mastery, and quality of life in a recent feasibility trial [29]. Other compelling grounds for the adaptation of PiB include the fact that it was evaluated across multiple institutions with coaches of different backgrounds, indicating scope for alignment with existing NHS pathways for people with dementia and their caregivers; it could, for example, be delivered in NHS memory clinics, or third sector support hubs with coaches from the NHS or voluntary organizations, or tailored for delivery from primary care if service configurations included financial support. Finally, PiB was developed using the Medical Research Council framework for complex interventions [30] and designed in partnership with caregivers and professionals [30-33]. These criteria suggested PiB offered more scope for investment and adaptation than the START family caregiver intervention trialed recently in the United Kingdom, but which reported smaller statistical differences on depression scales and did not set out to evaluate implementation or access strategies [34-37].

To aid implementation in the United Kingdom, the authors judged there was a clear need for adaptation of PiB content. For example, PiB videos made frequent reference to services provided in the Netherlands. References to the Netherlands towns and services also occurred in case studies and text summaries in each module. The authors determined these could limit effectiveness and provided clear grounds for adaptation involving UK caregivers and key stakeholders.

**Figure 1:**
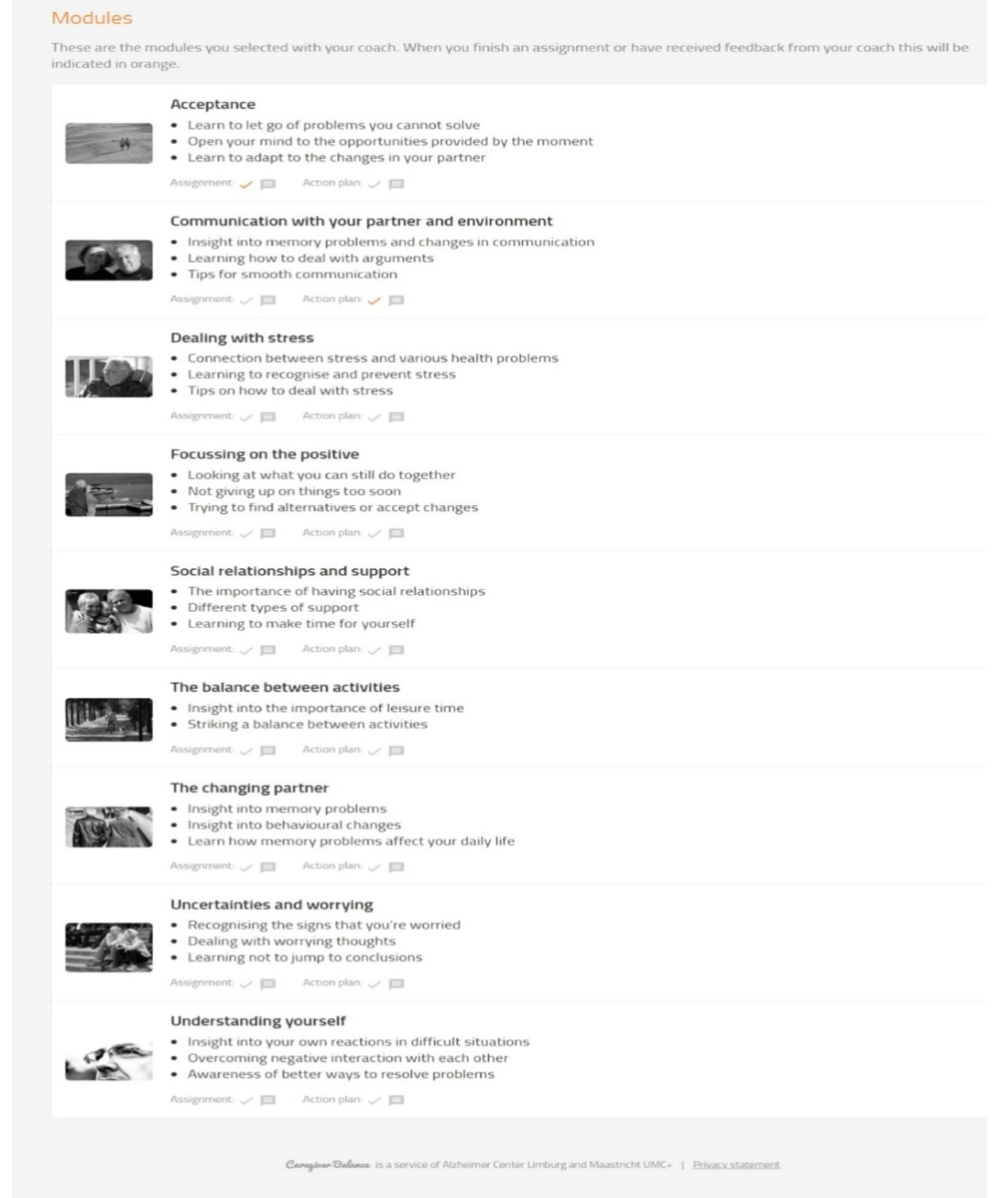
Partner in Balance (PiB) Module menu page [28].

PiB uses Social Learning Theory [37] and the stress and coping paradigm [38] to bolster resilience and equip caregivers with problem-solving and coping skills before disease progression when caring may become more difficult [39,40]. PiB blends coaching with web-based learning, with caregivers selecting from 9 stand-alone modules see Figure 1).

**Figure 2:**
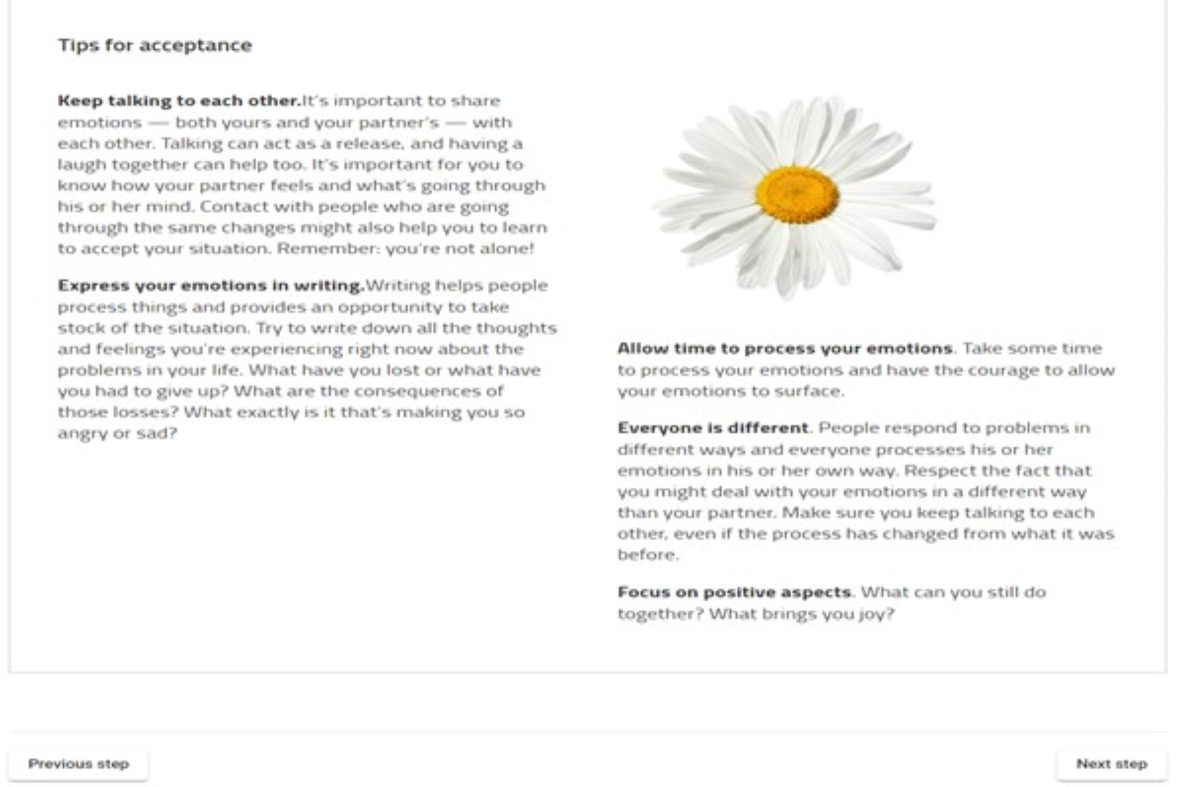
Partner in Balance (PiB) tips explanation page (Acceptance Module) [28].

**Figure 3:**
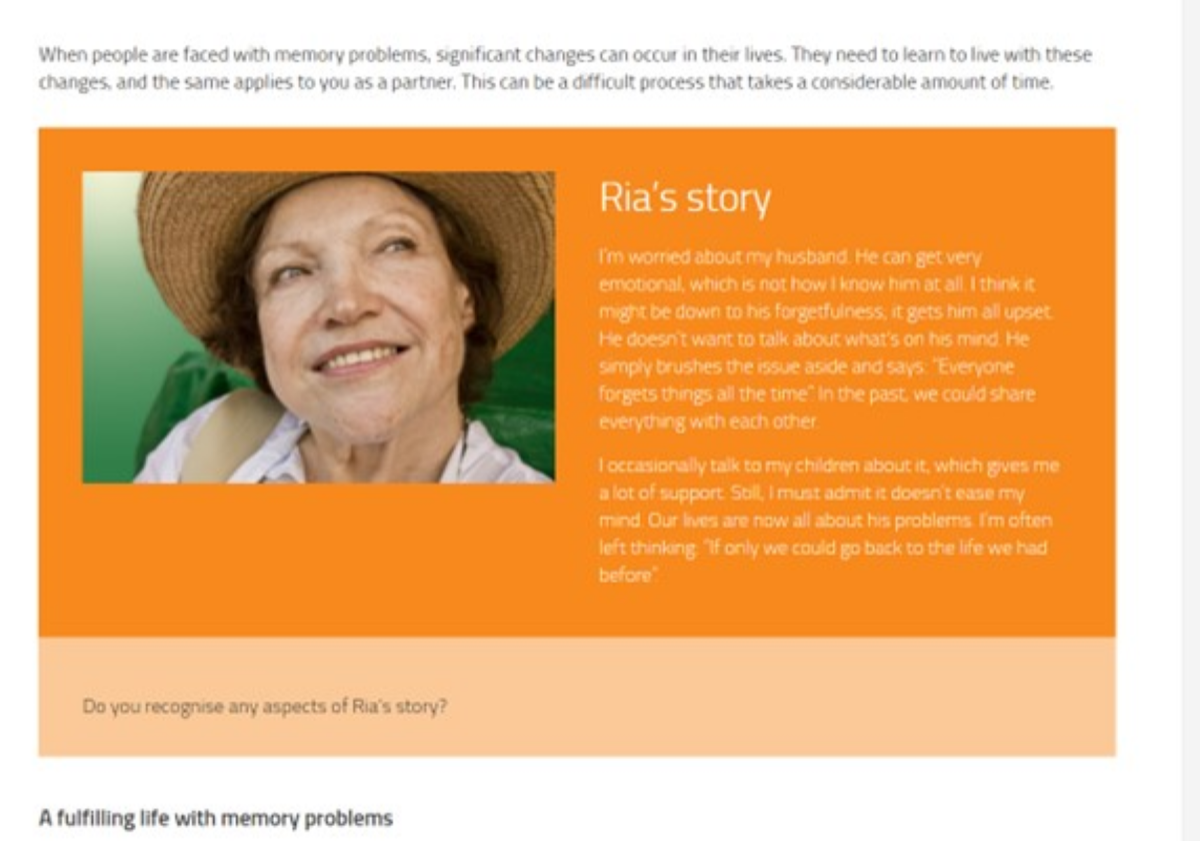
Case study Partner in Balance (PiB) explanation page (Acceptance Module) [28].

Each module provides text-based psycho-education, supported by a video vignette of caregivers sharing experiences, reinforced by practical tips, self-reflection, and a step-by-step change plan [28] (see Figures 2 and 3). Caregivers are supported by a coach (psychologist) to choose 3 or 4 modules that address their specific needs. A step-by-step plan is repeated across modules to establish systematic thinking and goal setting, working through barriers and solutions to develop self-empowerment, and better management of challenging situations [32]. Caregivers are encouraged to contact the coach for support at any point during the use of the program using the embedded email facility or telephone. A final meeting with the coach reflects on progress and consolidates learning.

## Methods

### Research Questions and Objectives

Working with caregivers and health care staff we aimed to identify what features of PiB required adaptation and cocreate modifications to achieve wide engagement among caregivers in the United Kingdom.

### Study Design

Co-design methods were used for the adaptation of PiB as they offer a robust, adaptable means of harnessing multiple-user perspectives to cocreate solutions [41] and are particularly suited to the challenges of research aimed at the cultural adaptation of existing interventions [42,43]. They have been successfully applied previously in the development of educational interventions aimed at caregivers [44]. Because PiB provided readymade trigger material for use in the interview setting, we opted to use Accelerated Experience-Based Co-Design (AEBCD) [45].

### Theoretical Approach

**Figure 4:**
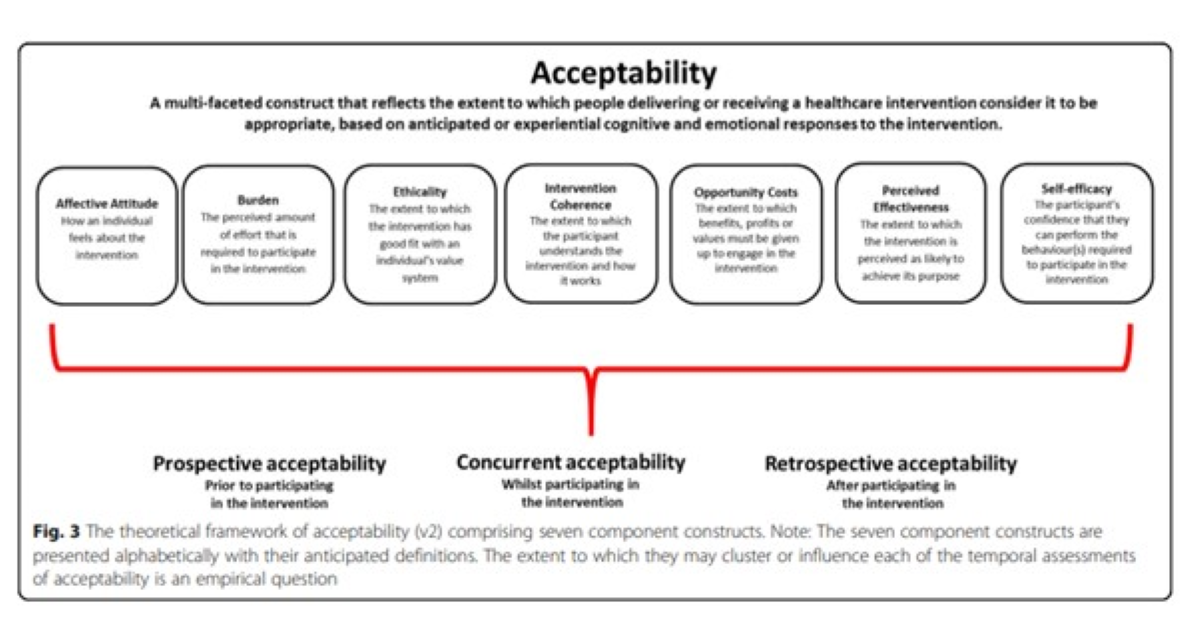
Theoretical framework of acceptability. Reproduced from [49] which is published under Creative Commons Attribution 4.0 International License [51]" .

Experience-based co-design works with concepts of “Emotional Touchpoints” [46,47]. We operationalized these as “reactions containing a feeling or cognitive statement, about text, instructions, content, or image, that worked well or caused concern” [48]. Working from this conceptual base we drew on the Theoretical Framework of Acceptability [49] (TFA; see Figure 4) to link emotional touchpoints to the components of PiB that caregivers and staff experienced as either acceptable or not acceptable. A similar approach was used for adapting coaching interventions aimed at managing diabetes [50]. The TFA [49] conceptualizes acceptability as a construct with multiple emotional and cognitive facets spanning affective attitude, burden, perceived effectiveness, ethicality, intervention coherence, opportunity costs, and self-efficacy.

### Procedure

Our implementation of AEBCD had three phases: (1) interviews with caregivers and staff to co-design adaptations, (2) workshops with caregivers and staff to debate the redesign modifications, and (3) consultations to refine and check the integrity of final adaptations. Results from these research phases were taken forward to build modifications to PiB. We describe these stages in detail below following an outline of the recruitment process.

### Sampling and Recruitment of Staff and Caregivers for Interviews and Workshops

Prospective participants (family caregivers and staff) were referred to the study by research staff based at Clinical Research Networks across 3 UK areas (Bradford, Nottingham, and Norfolk). All participants were screened for eligibility; to be included, staff were required to have a “main work role supporting people caring for people living with dementia.” Family caregivers needed to be 18 years old or above, able to communicate in English, have a first-degree relationship (eg, spouse, partner, sibling, daughter, or son) with the person cared for, and be currently caring or have been caring within the last 12 months. All participants were required to have the capacity to give informed consent, where possible recruitment aimed to achieve a diversity of people by gender, ethnicity, age, and role.

Potential participants were referred to the study by regional research staff who screened for eligibility and obtained permission to contact. Follow-up calls by the study Research Associate (RA) screened participants to ensure a mix of sex, age, and role were recruited. Very few (n=3) potential participants came forward from ethnic groups in the regions. To boost recruitment, we undertook a consultation exercise in a dementia support group working with a local community leader who facilitated translation in Bradford. The consultation uncovered strong reservations about being filmed in the interview setting. Other reasons for declining to take part were a lack of interest in web-based learning, preference for family/face-to-face peer group learning, and fear or hesitancy about discussing dementia prevalence due to stigma regarding dementia in the community. Time limits set by study progress targets did not allow further steps to improve recruitment from ethnic groups.

After screening for eligibility and providing permission to be contacted, potential participants (both caregivers and staff) were sent a study information pack and consent form by the study RA. Once consented, participants were recontacted by the RA for further screening to check interest and eligibility, and once confirmed, booked into an interview or a workshop.

### Phase 1: Interviews

Interviews using PiB as “trigger material” in accordance with AEBCD took place between December 2021 and May 2022. A topic guide was used (Multimedia Appendix 1) to check responses to format, content, images, and language and an open conversational approach was maintained to allow participants time to develop “emotional touchpoints” in response to PiB materials. The “think aloud method” [52] was used to encourage caregivers and staff to voice their reactions and empower caregivers in the redesign of PiB. Reflexive notes were completed by the RA after each interview and used as methodological support to the participatory process of the AEBCD process and the “think aloud” method [53]. Interviews were audio-recorded, transcribed, and areas of ambiguity in the text were checked and verified by the RA against video and audio material.

### Selection of Data for Review in Workshops

Interview transcripts were subjected to content coding. Data detailing redesign modifications were identified and imported into an Excel (Microsoft Corp) spreadsheet according to the item they related to, following standard content analysis methods [54]. Data were marked up to show where participants expressed a positive, neutral, or negative opinion relating to the PiB component in an approach approximating sentiment analysis, which aims to identify underlying emotional components of language (whether written or spoken), with a focus on classifying positive or negative opinions [55]. Core redesign proposals were selected from the Excel matrix by 3 researchers (FS, FG, and JC) and representative samples of the audio and transcript data were cut and embedded in a PowerPoint (Microsoft Corp) presentation for debate and review in workshops.

### Phase 2: Workshops

Workshops (convened in June 2022) aimed at confirmation, problem-solving, and expansion of the co-design ideas from the interviews. Representative proposals for the redesign of PiB were shared during the workshops to achieve agreement on the final modification. The conversation was free-ranging around the redesign proposals without the use of additional prompts. Any new modifications raised at the workshop stage were incorporated into the redesign proposals. Data summaries were discussed by the research team.

### Phase 3: Stakeholder Consultation and Working Party Adjudication

Input from the study service user advisory group (SUAG) was sought to review, refine, and confirm the deliberative decision-making work of the workshops and ensure the clarity and feasibility of the co-designed recommendations. Where areas of disagreement were still outstanding, the team took further advice from an Adaptation Working Party, established in line with recommendations for the adaptation of complex interventions [56]. The Adaptation Working Party comprised 6 experts from the wider program management group (PMG; clinical psychologists and third sector champions) and stakeholders from Dementia UK and Together in Dementia Everyday. A final check on the psychological underpinnings of proposed adaptations was made by the clinical psychologists who had created PiB as partners in the study. All changes were approved by the wider PMG.

### Ethical Considerations

Ethical approval was granted by the Health Research Authority and Health and Care Research Wales, IRAS project (ID: 297595: Protocol number: 1. REC reference: 21/PR/1353: Sponsor University of Exeter).

## Results

### Phase 1: Interviews

Thirty-three participants (16 caregivers and 17 staff) took part in the interviews (see Table 1 for details). Six caregivers were in full-time paid work alongside their unpaid caring role and 10 caregivers were retired. Most caregivers were White females aged 47 to 83 years. Staff were drawn from a range of occupational roles as follows: admiral Nurse (n=2), community mental health nurse (n=8), clinical psychologist (n=2), memory service nurse (n=1), and community support worker (n=3). Most were female, White, aged 30-65 years, and had been in their posts for between 1 and 20 years.

**Table 1:** Sex, age, and ethnic profile of interview participants.

				Caregiver (n=16), n		Staff (n=17), n			Total (N=33), n
**Sex**									
	Male		7		7			14	
	Female		9		10			19	
**Age range (years)**									
	**>65**								7
		Male	4		1		5		
		Female	2		—^a^		2		
	**51-65**								17
		Male	3		2		5		
		Female	7		5		12		
	**41-50**								6
		Male	N/A^b^		3		3		
		Female	N/A		3		3		
	**30-40**								3
		Male	N/A		1		1		
		Female	N/A		2		2		
**Ethnicity**									
	White British		15		13			28	
	White Polish		N/A		1			1	
	Mixed race		N/A		1			1	
	Asian/British		N/A		1			1	
	Asian		N/A		1			1	
	Black		1		N/A			1	

^a^Not available.

^b^N/A: not applicable.

### Phase 2: Workshops

Thirty participants (13 caregivers and 17 staff) took part in the workshops, of which 11 of the caregivers took part in the interviews. Six workshops were undertaken: 5 web-based and 1 face-to-face at a Dementia Café. Workshops with 2-20 people lasted 30 to 45 minutes. They were audio-recorded and transcribed, and 5 were video-recorded. Participants were predominantly White, female, and under 65 years (Table 2). Ten participants were retired and caring full-time, and 3 participants were in full-time paid work alongside caring. Thirteen of the staff were new to the study and were employed as dementia care support workers, 4 (an occupational therapist, a health care support worker, and 2 community mental health nurses) staff had taken part in interviews.

**Table 2:** Sex, age, and ethnic profile of workshop participants.

		Caregiver (n=13), n	Staff (n=17), n	Total (N=30), n
**Sex**				
	Female	7	15	22
	Male	6	2	8
**Age (years)**				
	<65	3	16	19
	>65	10	1	11
**Ethnicity**				
	White	11	15	26
	Black	1	1	2
	Others	1	1	2

### Concurrent Acceptability of Caregivers and Staff Toward PiB Content (Words and Imagery)

Most caregivers found PiB acceptable and were generally inspired by the PiB’s offer of an opportunity to learn and share, with some indicating that they found the content of the modules “extremely stimulating” [01-04C, male caring for partner less than 6 months] and the videos “very powerful [going] through the whole gamut of emotions that you know, the anger, the communication, the acceptance, the optimism. I really like that” [01-19C, male caregiver, wife newly diagnosed within 1 month]. Caregivers were also motivated by the possibilities of new learning which could help them cope and “just get better and better [...] and less stressed” [03-13C, female, caring for husband for 4 years]. The content was experienced as “uplifting” [01-22C, female caring for husband and mother for five years].

A minority (4 caregivers) were not inspired and found PiB unacceptable because the content and format did not suit their preferences, coping styles, or situations*.* For example, caregiver 01-17C experienced PiB’s grouping of common issues as irreconcilable with his preference for personalized [11] individual support: “This [PiB] is like frequently asked questions! That’s the concept of it” [01-17C, male, caring for wife for six months]. Another (caregiver, 01-29C) could not respond to PiB’s invitation to plan for the future because her care situation was too challenging.

It’s like looking at a medical book, [...] Having a list is not good, because it’s giving ideas of what’s wrong, [...] it could be counterproductive because my husband isn’t physically well, [...] I’m not going to achieve anything.

01-18C, female caring for husband for 4 years

Most participants found the PiB “Top-Tips” and video storylines useful and informative. However, not all caregivers and staff experienced the video content as culturally appropriate, and some reported they could not read the language or follow instructions easily. These difficulties are featured strongly in recommendations for redesign proposals. Our content analysis drew these areas into three themes: (1) video narration and imagery, (2) complex language, and (3) use of inclusive wording. We elaborate on these below highlighting where our findings link with the core constructs of the TFA.

### Video Narration and Imagery: Create New Videos Narrated in English Depicting Less Polished Contexts

Caregivers found it difficult to (1) locate and operate a button provided below the video screen that switched on the English subtitles and (2) read the small subtitle text. Most caregivers generally preferred the idea of English narration in the video content. These issues link with the TFA construct of “Burden” which draws attention to the amount of effort a participant needs to put into use an intervention (Figure 4).

Caregiver: I think I would definitely prefer it in English, [...] and, especially, on a screen which is quite small, and I wear glasses.

02-03C female, caring for mother

Caregivers and staff also reacted against the “polished” environments depicted in the videos. These could encourage negative self-comparisons which correspond with the TFA Self-Efficacy construct.

I felt that I wasn’t doing as well as [the women in the videos]. They were so well groomed tidy and smart, and the rooms were all perfect! Sometimes, I do not get to comb my hair!

01-13C, female, caring for husband with severe dementia

Staff were particularly concerned about how the subtitles and Dutch narrative placed distance between participants and the message being conveyed. This included family caregivers from ethnic backgrounds who could understand the spoken word but might not read English. They saw this as compromising learning and the effectiveness of the intervention.

Some people might not be reading English – due to it not being their first language or due to poor reading skills. These people would understand if English was spoken in the videos.

Staff, 02-02S

The use of subtitles risked undermining the effectiveness of PiB as caregivers could be distracted by the emotional inflection of the moving image and miss the details of the written content.

Some people might miss the words on the subtitles because they are looking at the video so they would miss the message.

02-01S

When initial subtitles come up in Dutch introducing the speaker and then the English subtitles are overlaid on this, it can be quite confusing

staff, 01-05S

A concern was also raised about the intervention videos which drew comment from staff on their lack of diversity in their representation of family caregivers.

‘They [the caregivers shown in the videos] are all white women!’ If I were a male carer [...] I might feel slightly alienated [...]. So, the fact that they are not represented is not particularly ideal.

staff 01-14S

### Complex Language: Simplify the Language and Shape it to Reflect Everyday English

Concern about complex wording was a common theme in caregivers’ and staffs’ reactions to PiB and redesign recommendations. These connect with the TFA dimensions of intervention: Burden, Coherence, Self-Efficacy, and Effectiveness. Some struggled to interpret the step-by-step instructions in PiB Action Plans.

Caregiver: So, [...] I think the obstacles bit is step three [...] You know, [...] it's like, ‘In case blah blah, you need to return to step one, once you've done that you need to go step two and three, and then step four, so this is quite complex.

01-01C, female, caring for mother

Interviewer’s reflexive notes (01-04C reviewing Acceptance module): When he reached the Assignment section [...] he switched back and forth from the Assignment to the Action plan and said he found it lacked coherence or clarity. He said [...] ‘It's a little bit confusing to me'. [...]. He thought it implied taking a 'SWOT analysis' approach to life’s problems which did not appeal to him. [He said] ' the thing [...] scares me a little bit!’.

Reflexive notes dated 4.1.2022

Caregivers did not like the formal terms used in PiB such as “Assignment,” “Action plan,” and “Goal.” Staff was concerned that this language was “complex.” Wording simplification was suggested to reduce the effort spent in attempting to interpret incomprehensible wording that some attributed to the Dutch-to-English translation.

Not a very happy translation is [it] ‘Communication with your environment’ I think [they] mean with your, your family.

01-20C, male, caring for wife

This is not the language of everyday! Nobody on the street uses the word ‘social relationships’, people talk about their family and friends!

staff 01-07S

### Use of Inclusive Wording Throughout to Reflect the Diversity of Caring Roles

Comparing our data with the “Affective Attitudes” construct of the TFA, we found that daughter-carer participants were influenced by their dissatisfaction with the word “Partner” being used throughout the PiB resource to address caregivers because it seemed to exclude the experience of adult children as caregivers.

What I struggle with a little bit is you don't expect ‘to parent a parent’. I think [this will need] some tailoring depending on whether it's partner or whether it's the child, caring for the parent kind of thing.

01-01C, female, caring for mother for two years

This extended to the “Top-Tips” section which offered advice to older couples living together, which also left daughters feeling excluded:

Mum’s not a partner, but there's one of those big sections just [...], after the daisy where it says ‘Keep talking to each other’. By and large [...] it's mostly... targeted at partners

01-23C, female caring for mother for over 12 months

Staff were concerned that the word partner did not include children or grandchildren who are often involved in familial care “There isn’t anything about young children in here” [01:14, Staff]. Caregivers held the same view “I would say it needed to be appropriate for people's children certainly” [01-16C, female caring for mother for over 3 years]. Finally, the meaning of the word “partner” was not clear for all older married male caregivers:

Caregiver: ‘Huh just partner in balance. Yeah?

Interviewer: So, it's not very clear for you, that phrase then Partner in balance?

Caregiver: Not exceptionally no. Okay yeah’ partner in balance’ hmm it does it does make sense, I suppose, I still thought about it a bit longer

01-12C, male husband caring for wife for more than 12 months

### Summary of Co-Designed Adaptations to PiB Imagery and Wording

The foremost recommendation was to create new videos for each of the 9 modules of PiB to show a more diverse population of caregivers, speaking in English in neutral environments and dispensing with subtitles. The second was to ensure the use of inclusive language throughout the resource to engage all caregivers and replace the word “partner” with “Relative/ friend / partner.” The third was to simplify wording throughout to improve comprehension and engagement. The final adaptation was to revise all “Top-Tips” and case studies to incorporate experiences of “non-partner” caring relationships and replace place names and first names with English.

### Stage 2: Workshop Deliberation

During the workshop, participants were presented with redesigned wording (see Table 3) and proposals for the creation of new videos. A consensus was reached on the need to film new videos using caregivers speaking English. The research team approved the need to create new videos with English-speaking caregivers in the United Kingdom. The production of new videos was taken forward and these were produced on location at 2 of the collaborating Universities (Universities of East Anglia and Bradford). Their content was guided by the interview sheet that had been used by the creators of PiB. As far as possible, caregivers were recruited who were diverse in age, gender, and culture.

Workshop feedback also helped to improve and refine the wording (vocabulary, tone, terms used, and appropriate translations). However, no consensus was achieved regarding the simplification of language used in the Assignment and Action Plan sections of PiB.

**Table 3:** Alternative wording development for Partner in Balance.

Original PiB^a^ wording modules and sections	Proposed new wording
“Social Relationship and support”	“Connecting with family and friends”
“Communication with your partner and environment”	“Communicating with others” “Talking and sharing” “Keeping in touch with others”
“Assignment”	“How am I?” “How am I doing?” “My journal review”
“Action plan”	“What I can do” “My plan” “My next steps” “My support plan”
“Partner”	“Person” “Relative/family member/friend”

^a^PiB: Partner in Balance.

### Stage 3: Service User Advisory Group and Working Party Adjudication

To move forward with wording simplification, the research team drew on the expertise of the SUAG and the Adaptation Working Party to adjudicate and decide on the final wording redesign. This process was followed as per the recommendations set out in ADAPT guidelines [56] and achieved a consensus on new wording (see Table 4).

The alternative wording combinations approved by the SUAG and Adaptation Working Party respected the key emphasis put forward by caregivers in the “think aloud” interviews insofar as they prioritized everyday simplicity and inclusivity of caregiver roles. The process and stages of wording development are listed in Table 5. All changes were approved by the CareCoach PMG.

**Table 4:** Final modified wording.

Original PiB^a^ wording modules and sections	New wording
“Social Relationship and support”	“Building my support”
“Communication with your partner and environment”	“Communication”
“Assignment”	“How am I doing”
“Action plan”	“My next steps”
“Partner”	“Relative/family member/friend”

^a^PiB: Partner in Balance.

**Table 5:** List co-design and review of wording modifications.

Co-design stages 1-5	Chronology of wording co-design			
Stage 1: Module and section header wording identified as problematic during interviews (December 2021-April 2022).	Module header “Communication with your partner and environment” (module 2 in the PiB^a,b^)	Module header “Social Relationship and support” (module 5 in the PiB)	Section 4 header (common to all 9 modules) “Assignment”	Section 5 header common to all 9 modules) “Action plan”
Stage 2: Alternative wording suggested by participants during interviews (December 2021-April 2022).	Communicating with others Talking and sharing Keeping in touch with others	Connecting with family and friends	How am I? How am I doing? My journal review	What I can do My plan My next steps My support plan
Stage 3: Alternative wording suggested in workshops by participants:	Communicating with and listening to each other Communicating with and listening to your partner Communicating with my Partner	Your support network Your community and family	How am I doing? How am I? How do I solve the problem/know the issues? Get some support?	What can help me? Moving forward Looking forward
Stage 4: Wording adjudication review June 2022 SUAG^c^ participants (Supported by the research team)	Talking and sharing Talking listening and sharing Sharing with family and friends Sharing with others Communication	Building support around you Building support up Building your support network	How am I doing? Reflection	My next steps
Stage 5: Working Party adjudication meeting June 2022	Confirms “Communication”	Confirms “Building your support”	Confirms “How am I doing”	Confirms “My next steps”
Final wording taken forward	Communication	Building your support^c^	How am I doing^d^	My next steps

^a^PiB: Partner in Balance.

^b^ Module headers use “Your” which gives ownership in context of self-directed nature of PiB learning.

^c^ SUAG: service user advisory group.

^d^Section headers use “I” and “My”, gives ownership.

## Discussion

### Principal Results

The family caregivers and staff involved in the co-design process reported in this study were motivated by a preference for familiar-sounding accessible language and video films that reflected their cultural identities. They prioritized making changes that improved comprehension and feeling validated in imagery. The fragments of Dutch cultural imagery and language that remained in the English version of PiB were experienced as barriers to UK caregivers’ engagement and new videos were created and incorporated into CareCoach to maximize acceptability. Daughter-caregivers prioritized inclusive wording that accommodated their role, as they could not identify with the word “partner.”

Our work adapting PiB drew on the insights of the TFA model of acceptability [49], which provides a system for evaluating the extent to which a given intervention has a good fit with an individual’s value system. It is worth noting that while the caregivers and staff we sampled were predominantly White, they championed the need to ensure inclusivity.

Ultimately our findings underscore the importance of using diverse imagery and language so that caregivers can see themselves, their cared-for relative with dementia, and the wider community in which they live in the materials being presented to them. Triggers for adaptation arose when caregivers and staff encountered images that did not fit with their own or wider community social/cultural identity, consonant with a negative Affective Attitude and Ethicality dimensions of the TFA model of acceptability and the Evidence-Based Co-Design method [41,49]. These data demonstrate the fundamental importance of adaptation work to improve the inclusivity of interventions ahead of implementation to maximize service user engagement.

Directly including unpaid family caregivers in the adaptation of the PiB blended web-based coaching support program is a key strength of this research process. We strengthened the rigor and quality of data interpretation by using reflexive field notes (written after interviews) and used “constant comparison” during interviews to examine meanings. Research team meetings contributed to reflexive discussion and to reviewing data by iteration. The meetings provided a space where issues under analytical focus could be bracketed. Meeting notes created an audit trail of steps in this process. These checks align with techniques for improving confirmability [57].

A further strength of this study is how it applies established guidelines [56] to report on adapting the PiB for the UK context. Comparing data items against the TFA of health care interventions [49] added interpretative rigor to data analysis.

### Limitations

A weakness of this study is the lower rates of recruitment of caregivers from diverse ethnic populations. This is a common challenge in studies using web-based methods and internet-based interventions [58,59] and future research needs to address this. For example, future studies could adopt flexible recruitment approaches to allow fieldwork to take place in community settings where there is access to public computers to better include participants from marginalized communities. This pathway was not open in this study due to COVID-19 restrictions.

### Comparison With Prior Work

Our findings support previous research showing that interventions require cultural tailoring to match the cultural identities of the target community [20,21,60]. Our findings confirm that language used in complex interventions needs to be straightforward, so as not to overburden users and those tasked with supporting them (here, coaches) [24-27].

### Conclusions

In this study, 2 rounds of consultation were insufficient to achieve agreement on simplifying wording. A final decision on changes therefore had to be adjudicated by the working party established to facilitate this aspect. An important insight this study established for co-design aimed at adapting interventions from other contexts is that the design process involves layers of negotiation, and requires compromises to reach a final consensus on making adaptations.

The AEBCD approach helped prioritize the voices and presence of caregivers in adapting the PiB intervention for CareCoach. Interviews using PiB as trigger material worked effectively and cost-effectively as a means to gather the views of family caregivers and staff conveniently. The CareCoach program addresses the challenge set by the World Health Organization [61] for 75% of countries to provide support and training programs for caregivers and families of people with dementia by 2025 [62]. The robust AEBCD approach has significantly contributed to ensuring that the adapted intervention provides resources that can be tailored to the needs of UK caregivers and has real potential to improve their knowledge and skills in caregiving and communication and to build caregivers’ ability to cope with the stresses of caring in dementia in the United Kingdom.

## Appendix

Multimedia Appendix 1 Interviewer Topic Guide Interviews Stage 1.

## Abbreviations

AEBCD: Accelerated Experience-Based Co-Design
PiB: Partner in Balance
PMG: program management group
RA: Research Associate
SUAG: service user advisory group
TFA: Theoretical Framework of Acceptability
