# Supplementary material for: Adapting a Dutch Web-Based Intervention to Support Family Caregivers of People With Dementia in the UK Context: Accelerated Experience-Based Co-Design
Source: JMIR Form Res. 2024 May 22;8:e52389. doi: 10.2196/52389 (PMC11153978; doi:10.2196/52389)
Supplement: Multimedia Appendix 1 [file formative_v8i1e52389_app1.docx]

**Appendix A**

**Interviewer Topic Guide Interviews Stage 1**

Interviewer prompts (topic guide) aimed to focus the participant on how they experienced the design and function of PiB in terms of the structure (format) and the more nuanced *‘experience of and emotional response to’* video resources and wording:

1. *Content (images, words, philosophy, too little or too much information)*

*1.1 how accessible it is – can they understand it?*

*1.2 how they feel about it while they are using it.*

*1.3 how well it served its purpose, (does it inspire or motivate and communicate well?)*

1. *Mode of delivery (online app, working alone).*

*2.1 how well it fits into the context in which they are using it?*

1. *Format / layout (tone, colour, ease of navigation, length)*
